# Age estimation of children and adolescents from mandibles using machine learning

**DOI:** 10.1038/s41598-025-21221-0

**Published:** 2025-10-07

**Authors:** Erika Calvano Küchler, Paula Patrícia Krohn, Eduarda Gaspari Campos Efeiche, Livia Livia Alves Antunes, Allan Abuabara, Bianca Marques de Mattos de Araujo, Flares Baratto-Filho, Svenja Beisel-Memmert, Christian Kirschneck, Cristiano Miranda de Araujo

**Affiliations:** 1 Department of Orthodontics, MedicalFaculty, University Hospital Bonn, Welschnonnenstr. 17, 53111 Bonn, Germany; 2 University of Tuiuti of Paraná, Curitiba, Paraná Brazil; 3 Postgraduate Program in Dentistry, Health Institute of Nova Friburgo, Fluminense Federal University, Rio de Janeiro, Brazil; 4 University of the Joinville Region, Joinville, Santa Catarina Brazil

**Keywords:** Machine learning, Artificial intelligence, Age determination by skeleton, Mandible, Computational biology and bioinformatics, Medical research

## Abstract

Age estimation is a crucial step in forensic identification, particularly in scenarios where dental structures may be absent. This study aimed to develop and evaluate supervised machine learning models to predict chronological age based on mandibular morphometric measurements in children and adolescents. A sample of lateral cephalometric radiographs from 401 orthodontic patients aged between 6 and 16 years was analysed. Linear and angular mandibular measurements including the total mandibular length (Co-Pog), mandibular ramus height (Co-Go), mandibular body length (Go-Gn), and the gonial angle (Ar-Go-Me) were analysed. Eight supervised machine learning algorithms were trained to predict chronological age based on these measurements and sex. The dataset was split into training (80%) and test (20%) sets, with stratified 5-fold cross-validation to prevent overfitting. Model performance was evaluated using mean absolute error (MAE), mean squared error (MSE), root mean squared error (RMSE), and coefficient of determination (R²), with 95% confidence intervals estimated via bootstrapping. The models based on mandibular morphometric features and sex achieved a minimum MAE of 1.54 years (95% CI: 1.33–1.76) and RMSE of 1.93 (95% CI: 1.66–2.18) on the test set. Cross-validation confirmed model stability, with the Gradient Boosting Regressor achieving the best performance, showing a MAE of 1.21 (95% CI: 1.09–1.32) and R² of 0.56 (95% CI: 0.46–0.64). Total mandibular length (Co-Pog) and mandibular ramus height (Co-Go) were the most important predictors. Pairwise comparisons revealed statistically significant differences favoring ensemble methods over linear and simpler tree models. Supervised machine learning models demonstrated promising accuracy for age estimation based on mandibular measurements in growing individuals. Gradient Boosting emerged as the most effective algorithm. However, the generalizability of the models may be influenced by population-specific characteristics and the need for prior knowledge of certain predictor variables. Further external validations are recommended to enhance model applicability across diverse forensic contexts.

## Introduction

Forensic identification of unknown human remains fundamentally relies on the accurate estimation of biological characteristics, including age and sex. Age estimation, in particular, is critical in forensic medicine for both individual identification and mass disaster victim reconciliation^1–3^. Beyond postmortem applications, age estimation contributes to the assessment of legal maturity, supporting decisions on whether an individual should be prosecuted as a juvenile or an adult, an important factor that can influence the severity of criminal sentencing^4^.

The mandible is the most frequently recovered bone in human remains. It is sometimes the only bone available for post-mortem investigation^5^ and it is well suited for age estimation, as the mandible exhibits more pronounced development changes than other craniofacial bones^6^. Mandibular dimensions, particularly mandibular length, ramus height, and mandibular angle (gonial angle) have shown strong correlations with age in humans^7^.

Recent advancements in Artificial Intelligence (AI) have introduced novel methodologies to address limitations inherent in conventional forensic age estimation techniques. Machine learning, a subset of AI, offers robust capabilities for nonlinear data analysis^8^ and has significantly improved forensic age estimation by providing faster, more standardized, and objectively quantifiable evaluations. While AI-driven approaches have been applied to mandibular analyses for age estimation, existing studies predominantly focus on dental age methodologies. Although machine learning methods based on dental age stages demonstrate high accuracy, in some crime circumstances, the mandible may be found without teeth due to postmortem tooth loss, intentional avulsion, or environmental factors, making forensic identification more challenging.

Thus, in the present study, we investigated the application of mandibular dimensions with machine learning algorithms for age estimation in children and adolescents.

## Methods

### Study design

This cross-sectional observational study examined orthodontic records from children and adolescents enrolled in the orthodontic treatment at the Bonn University-Germany. This project was conducted in accordance with the Declaration of Helsinki and approved by the Human Ethics Committee (2024-252-BO). Informed consent was obtained from patients and their legal guardians.

### Participants

Orthodontic records, including cephalometric radiographs, were screened for this study. Participants included were aged between 6 and 16 years old. Inclusion criteria consisted of children and adolescents without underlying syndromes or congenital alterations. Individuals presenting one or more teeth missing bilaterally due to agenesis or extraction were excluded from the analysis.

### Mandibular size assessment

**Fig. 1:**
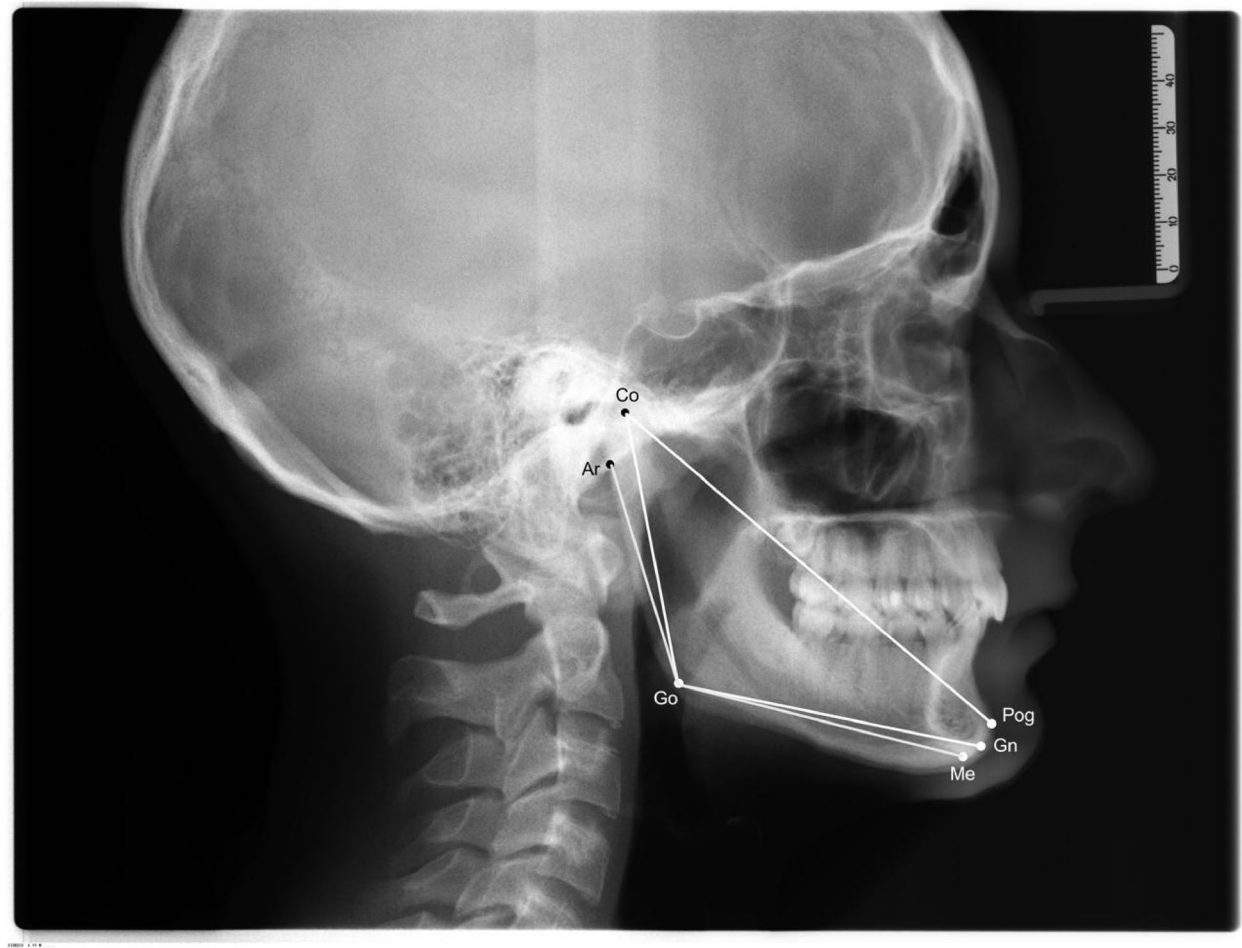
Lateral cephalometric radiograph showing anatomical landmarks. Linear measurements: Co-Pog, Co-Go, Go-Gn. Angular measurement: Ar-Go-Me. GN = Gnathion, ME = Menton (Me), Pog = Pogonion, Go = Gonion; Co = Condylion Ar = Articulare.

Digital pre-treatment lateral cephalometric radiographs were analyzed to evaluate mandibular dimensions. Radiographs were imported into OnyxCeph software (version 3.2.180; Image Instruments GmbH, Chemnitz, Germany) as lossless TIF files, calibrated, and digitally assessed. Each cephalogram was oriented using the Frankfort horizontal plane and the midsagittal reference line provided by the software, in order to minimize distortions caused by head inclination or rotation. The following anatomical landmarks were identified on each cephalograms (Fig. 1):

- Gnathion (Gn)—The most inferior and anterior point of the mandible at the midline.
- Menton (Me)—The lowest midline point of the chin.
- Pogonion (Pog)—The most anterior midline point of the chin.
- Gonion (Go)—The most posterior and inferior point of the mandibular angle (gonial angle).
- Condylion (Co)—The most superior point on the head of the mandibular condyle.
- Articulare (Ar)—The intersection point between the posterior border of the mandibular ramus and the base of the skull.

Based on the identified landmarks, the following measurements were recorded:

- Linear distances (mm):
  - Mandibular Ramus height: Co-Go Distance from Condylion (Co) to Gonion (Go).
  - Mandibular body length: Go-Gn - Distance from Gonion (Go) to Gnathion (Gn).
  - Total mandibular length: Co-Pog - Distance from Condylion (Co) to Pogonion (Pog).
- Angular measurement (°):
  - Mandibular angle (gonial angle): Ar-Go-Me—Gonial angle formed by the intersection of lines from Articulare (Ar) to Gonion (Go) and Gonion (Go) to Menton (Me). This angle was also considered a cephalometric indicator of vertical skeletal pattern, thereby incorporating potential vertical morphological influences into the modelling process.

To assess potential confounding effects of sagittal skeletal pattern on age estimation, the sample was classified into skeletal Classes I, II, and III based on the ANB angle, and chronological age was compared among these groups using one-way ANOVA.

### Sample size and power test

To assess the adequacy of the adopted sample size for each individual predictor, a post-hoc statistical power analysis was performed using G*Power software (version 3.1.9.6; Heinrich Heine University Düsseldorf, Germany; available at: https://www.psychologie.hhu.de/arbeitsgruppen/allgemeine-psychologie-und-arbeitspsychologie/gpower). For continuous predictors, the analysis was conducted under Exact - Correlation: Bivariate normal model, entering the observed Pearson correlation coefficient (*r*) with chronological age, α error probability, and total sample size. For the dichotomous predictor (sex), the analysis was performed under t tests—Means: Difference between two independent means [two groups], using the observed group means, standard deviations, and sample sizes. Given that this step was intended for exploratory variable selection prior to multivariable modelling, a more liberal significance level (α = 0.20) was adopted, as recommended by Hosmer, Lemeshow, and Sturdivant (2013)^33^, to reduce the likelihood of excluding potentially relevant predictors at the univariate stage.

### Feature selection

Predictor variables were selected based on their predictive ability concerning the dependent variable, chronological age. Initially, an exploratory analysis using Pearson correlation test was conducted to examine associations between each independent variable and chronological age. A significance level of 5% (α = 0.05) was adopted, and variables demonstrating statistically significant correlations (*p* < 0.05) were considered relevant and selected for inclusion in predictive models. This approach aimed to identify and retain only those variables that effectively contribute to the explanatory power of the models, thus reducing complexity and enhancing predictive efficiency.

### Model development

A predictive model was developed to estimate chronological age based on mandibular size and sex. To ensure a comprehensive evaluation of the relationships present in the dataset—from simple linear associations to more complex, nonlinear patterns—algorithms from four major categories of machine learning were carefully selected: linear models, tree-based models, instance-based methods, and artificial neural networks.

Specifically, eight algorithms were chosen:

- Linear Regression (LR), for its simplicity and interpretability.
- Gradient Boosting Regressor (GB), Random Forest Regressor (RF), Decision Tree Regressor (DT), and AdaBoost Regressor (ADA), representing tree-based models known for robustness and strong predictive capabilities.
- Support Vector Regression (SVR) and K-Nearest Neighbors Regressor (KNN), as instance-based methods effective in capturing complex nonlinear relationships.
- Multilayer Perceptron Regressor (MLP), an artificial neural network capable of identifying highly complex data patterns through multiple hidden layers.

This selection aimed to balance model interpretability, predictive performance, and adaptability to diverse data structures, allowing rigorous comparisons among the different methodological approaches^9–12^.

### Training, cross-validation, test and overfitting control

Prior to training the predictive models, the dataset was normalized to enhance numerical stability and improve algorithm learning efficiency. This procedure aims to reduce the influence of varying scales among variables, allowing each feature to contribute equally to model development.

The dataset was randomly split into training (80%) and testing (20%) sets. This approach ensured that model training was conducted on a substantial portion of the data, while an independent subset was reserved to evaluate the model’s ability to estimate chronological age in unseen individuals—simulating a real-world application.

To ensure greater generalization capability and minimize the risk of overfitting, stratified five-fold cross-validation was employed. This method involved splitting the data into five subsets, ensuring each subset maintained a representative distribution of the studied variables^13^. Each model was trained and validated repeatedly across these subsets, increasing robustness and reliability of performance metrics obtained.

### Hyperparameter optimization

Detailed hyperparameter optimization was performed using the Grid Search method, a systematic and exhaustive search for optimal parameter combinations. Hyperparameters define critical aspects of the algorithms’ learning process, directly impacting their capacity to detect patterns within the data^14^. Thus, this optimization aimed to identify parameter configurations that maximize the predictive performance of the models.

#### Model evaluation metrics and feature importance

The accuracy and reliability of predictions made by the developed machine learning models were assessed using the following performance metrics:

- Mean squared error (MSE): Measures the average of the squared differences between predicted and actual chronological ages, giving more weight to larger errors.
- Root mean squared error (RMSE): Calculated as the square root of the MSE, RMSE expresses prediction errors in the same unit (years) as chronological age, making model accuracy easier to interpret.
- Mean absolute error (MAE): Represents the average absolute magnitude of prediction errors, regardless of their direction. This metric provides a straightforward and objective measure of overall predictive accuracy.
- Coefficient of determination (R^2^): Indicates the percentage of total variability in chronological age that is explained by each predictive model. Higher values suggest greater explanatory power and more reliable predictions.

To accurately quantify the uncertainty surrounding each performance metric and assess prediction consistency, 95% confidence intervals (CI95%) were calculated using the bootstrap method with 1000 resampling iterations. This approach yielded robust variability estimates and reliable confidence intervals that reflect the models’ true performance. For cross-validation results, confidence intervals were derived from the distribution of metrics across different data folds, enabling a comprehensive evaluation of model stability and generalizability.

Visual representations of prediction errors were created using scatter plots, highlighting discrepancies between predicted and actual chronological ages. The prediction error plots were generated by overlaying scatter points on a semi-transparent bivariate kernel density heatmap, graded from navy blue to deep red to indicate the concentration of observations, with higher densities represented by colors closer to red.

Statistical comparisons of MAE values across models were also performed using bootstrap analysis. A statistically significant difference was identified when the computed confidence interval did not include zero, thereby confirming meaningful differences in predictive performance.

To evaluate the relative contribution of each input variable to the model’s predictions, feature importance scores were calculated using tools available in the scikit-learn library. These scores provide an estimate of the predictive weight of each variable and are typically derived from models that inherently support this type of analysis. For algorithms that do not natively offer this functionality—specifically K-Nearest Neighbors (KNN), Support Vector Regression (SVR), and Multilayer Perceptron (MLP)—feature importance analysis was not performed^15–19^.

**Fig. 2:**
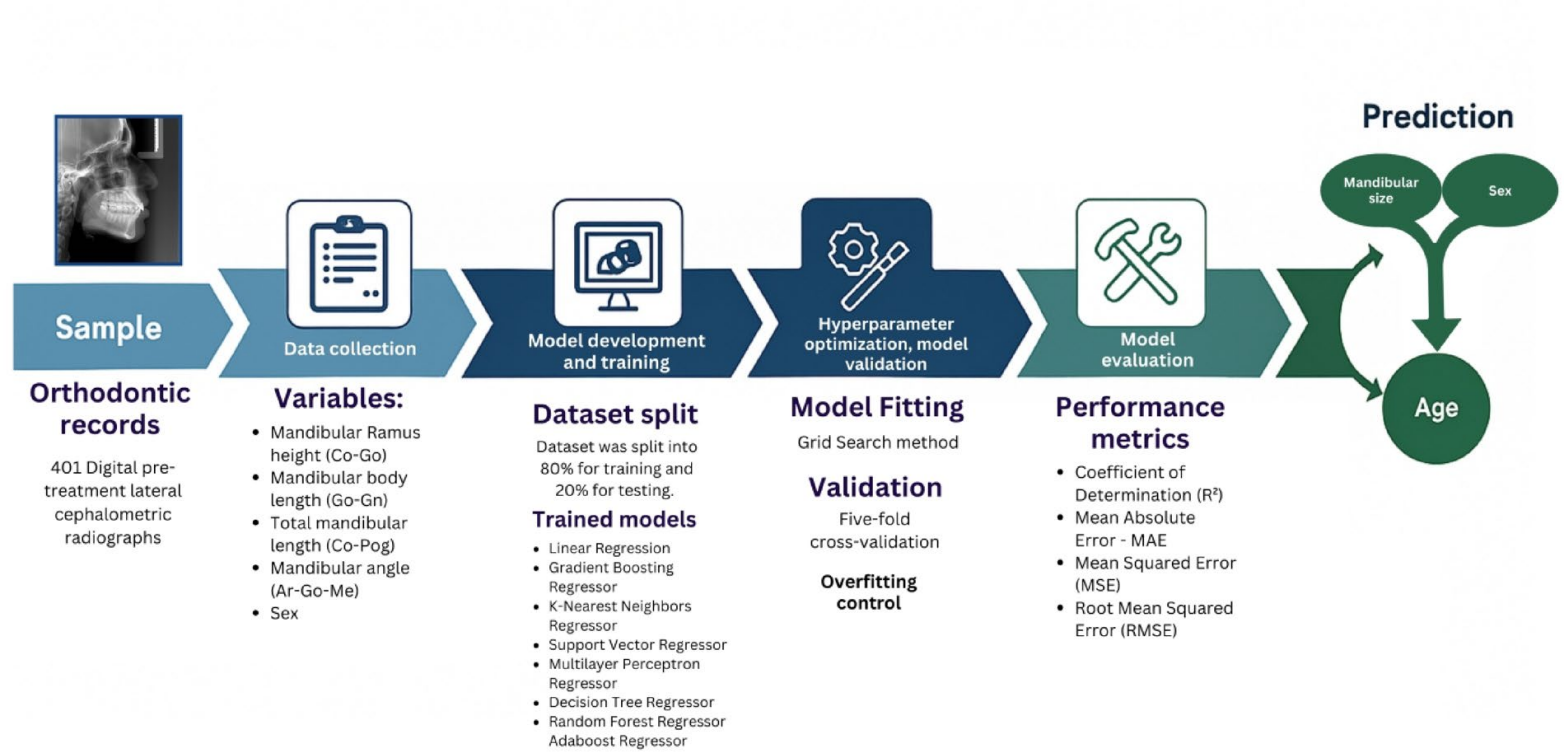
Flowchart of the main steps of the study.

All statistical analyses and visualizations were performed using the Python programming language in the Google Colaboratory environment. The entire workflow—comprising model construction, training, validation, hyperparameter optimization, and performance evaluation—is illustrated in Fig. 2. To ensure transparency and reproducibility, all scripts developed for the analysis are openly available at 10.5281/zenodo.15264847.

## Results

### Sample characterization

A total of 401 individuals were included, with an age ranging from 6.3 to 16.8 years old, and a mean age of 11.7 ± 2.3 years old; 200 (49.8%) were males and 201 (50.2%) were females (11.4 ± 2.38 years and 11.8 ± 2.37 years respectively). Post-hoc statistical power analysis demonstrated that all predictors assessed in the univariate stage achieved statistical power ≥ 80% for the observed associations, confirming both the adequacy of the sample size and sufficient predictive capacity to support their inclusion in the multivariable modelling. Sagittal skeletal classification (based on ANB) comprised 177 Class I, 150 Class II, and 74 Class III subjects. A one-way ANOVA comparing chronological age among these groups showed no statistically significant difference (*p* = 0.488), indicating a balanced age distribution across sagittal patterns.

### Model performance

**Fig. 3:**
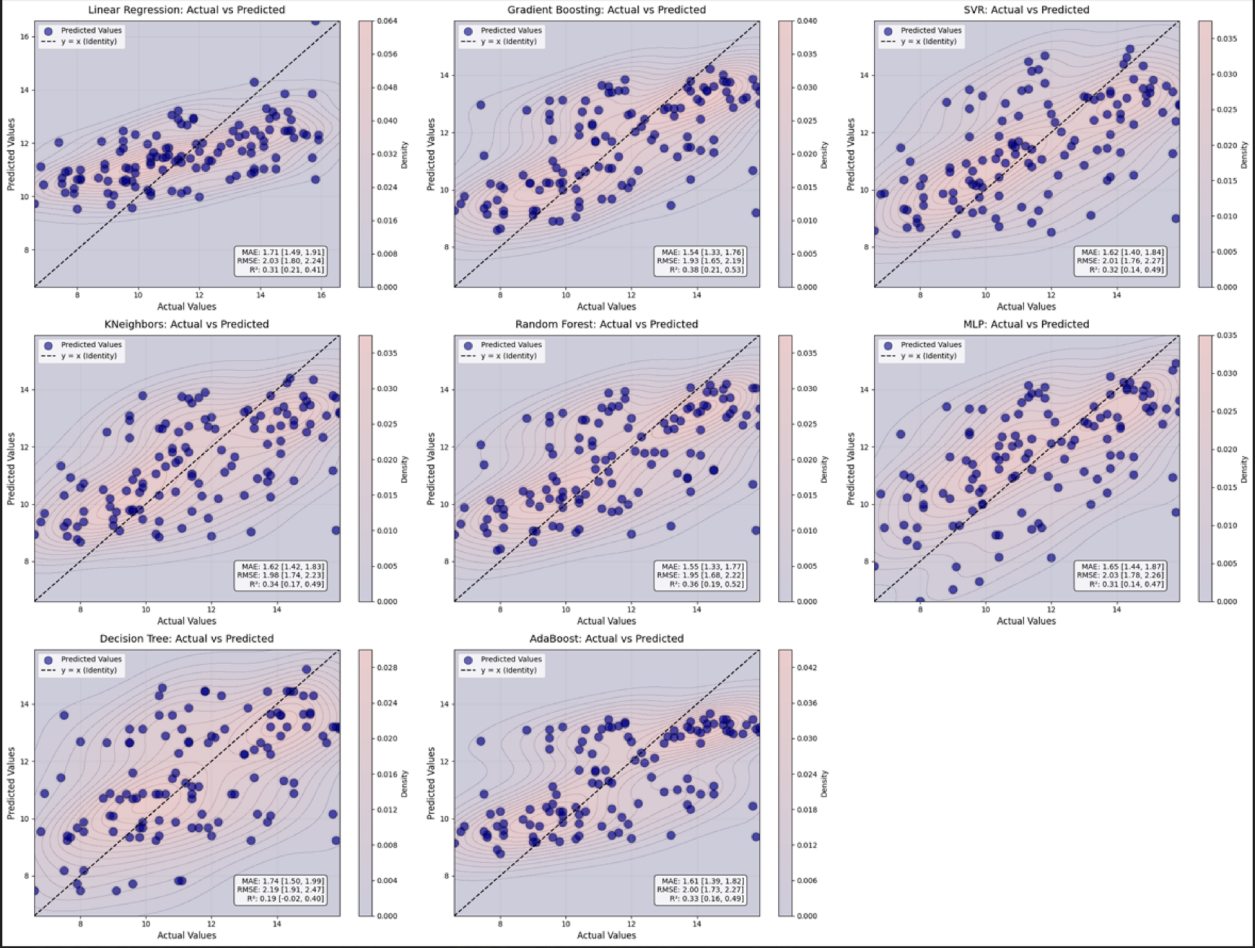
Scatter plots: Random Forest, Support Vector Machine (SVM), and K-Nearest Neighbors (KNN).

The predictive models based on mandibular morphometric features and sex demonstrated mean absolute errors (MAE) closer to 1.5 years when compared to actual chronological age. Among the evaluated algorithms, the Gradient Boosting model achieved the lowest MAE on the test set (1.54; 95% CI: 1.33–1.76), followed by the Random Forest, SVM (Support Vector Machine), and KNN (K-Nearest Neighbors) models (Fig. 3). Cross-validation supported this finding, with Gradient Boosting maintaining the lowest MAE (1.21; 95% CI 1.09–1.32) and the highest coefficient of determination (R^2^ = 0.56; 95% CI 0.46–0.64). However, R^2^ values in the test set indicated that only a moderate portion of the variability in chronological age was explained by the included predictors (R^2^ = 0.38; 95% CI 0.21–0.53) (Table 1).

**Table 1:** Summary of the metrics obtained during the cross-validation and testing phases of the regression models used to predict chronological age based on mandibular size and sex, along with their optimal hyperparameters.

Model	Optimal hyperparameters	Test data results [CI95%]	Cross-validation results [CI95%]
Gradient boosting	learning_rate: 0.1	MSE = 3.74 [2.73–4.81]	MSE = 2.37 [1.96–2.84]
	max_depth: 4	RMSE = 1.93 [1.65–2.19]	RMSE = 1.54 [1.40–1.69]
	min_samples_split: 2	R^2^ = 0.38 [0.21–0.53]	R^2^ = 0.56 [0.46–0.64]
	n_estimators: 100	MAE = 1.54 [1.33–1.76]	MAE = 1.21 [1.09–1.32]
Linear regression	fit_intercept: True	MSE = 4.12 [3.24–5.02]	MSE = 4.28 [2.64–6.07]
	copy_X: True	RMSE = 2.03 [1.80–2.24]	RMSE = 2.06 [1.62–2.46]
	n_jobs: -1	R^2^ = 0.31 [0.21–0.41]	R^2^ = 0.20 [-0.13–0.51]
	positive: True	MAE = 1.71 [1.49–1.91]	MAE = 1.56 [1.31–1.78]
Support vector machine	C: 1	MSE = 4.06 [3.09–5.16]	MSE = 2.84 [2.38–3.34]
	kernel: rbf	RMSE = 2.01 [1.76–2.27]	RMSE = 1.68 [1.54–1.83]
	degree: 2	R^2^ = 0.32 [0.14–0.49]	R^2^ = 0.47 [0.37–0.55]
		MAE = 1.62 [1.40–1.83]	MAE = 1.33 [1.20–1.47]
K-nearest neighbors	n_neighbors: 9	MSE = 3.94 [3.02–4.98]	MSE = 2.97 [2.45–3.55]
	p: 1	RMSE = 1.98 [1.74–2.23]	RMSE = 1.72 [1.57–1.88]
	weights: uniform	R^2^ = 0.34 [0.17–0.49]	R^2^ = 0.45 [0.34–0.54]
		MAE = 1.62 [1.42–1.83]	MAE = 1.37 [1.23–1.51]
Random forest	max_depth: None	MSE = 3.81 [2.81–4.92]	MSE = 2.29 [1.91–2.71]
	max_features: sqrt	RMSE = 1.95 [1.68–2.22]	RMSE = 1.51 [1.38–1.65]
	min_samples_leaf: 4	R^2^ = 0.36 [0.19–0.52]	R^2^ = 0.57 [0.48–0.65]
	min_samples_split: 10	MAE = 1.55 [1.33–1.77]	MAE = 1.18 [1.06–1.29]
	n_estimators: 200		
AdaBoost	learning_rate: 0.1	MSE = 4.01 [3.00–5.14]	MSE = 2.58 [2.17–3.02]
	loss: linear	RMSE = 2.00 [1.73–2.27]	RMSE = 1.60 [1.47–1.74]
	n_estimators: 100	R^2^ = 0.33 [0.16–0.49]	R^2^ = 0.52 [0.42–0.60]
		MAE = 1.61 [1.39–1.82]	MAE = 1.30 [1.19–1.42]
Decision tree	max_depth: 10	MSE = 4.83 [3.65–6.12]	MSE = 2.94 [2.31–3.65]
	max_features: sqrt	RMSE = 2.19 [1.91–2.47]	RMSE = 1.71 [1.52–1.91]
	min_samples_leaf: 4	R^2^ = 0.19 [-0.02–0.40]	R^2^ = 0.45 [0.31–0.57]
	min_samples_split: 10	MAE = 1.74 [1.50–1.99]	MAE = 1.29 [1.14–1.45]
MLP regressor	activation: relu	MSE = 4.11 [3.18–5.12]	MSE = 3.02 [2.52–3.65]
	alpha: 0.001	RMSE = 2.02 [1.78–2.26]	RMSE = 1.73 [1.59–1.91]
	hidden_layer_sizes: (100,)	R^2^ = 0.31 [0.14–0.47]	Rv = 0.44 [0.31–0.53]
	learning_rate: constant	MAE = 1.65 [1.44–1.87]	MAE = 1.40 [1.27–1.53]
	solver: adam		

### Pairwise model comparison

**Fig. 4:**
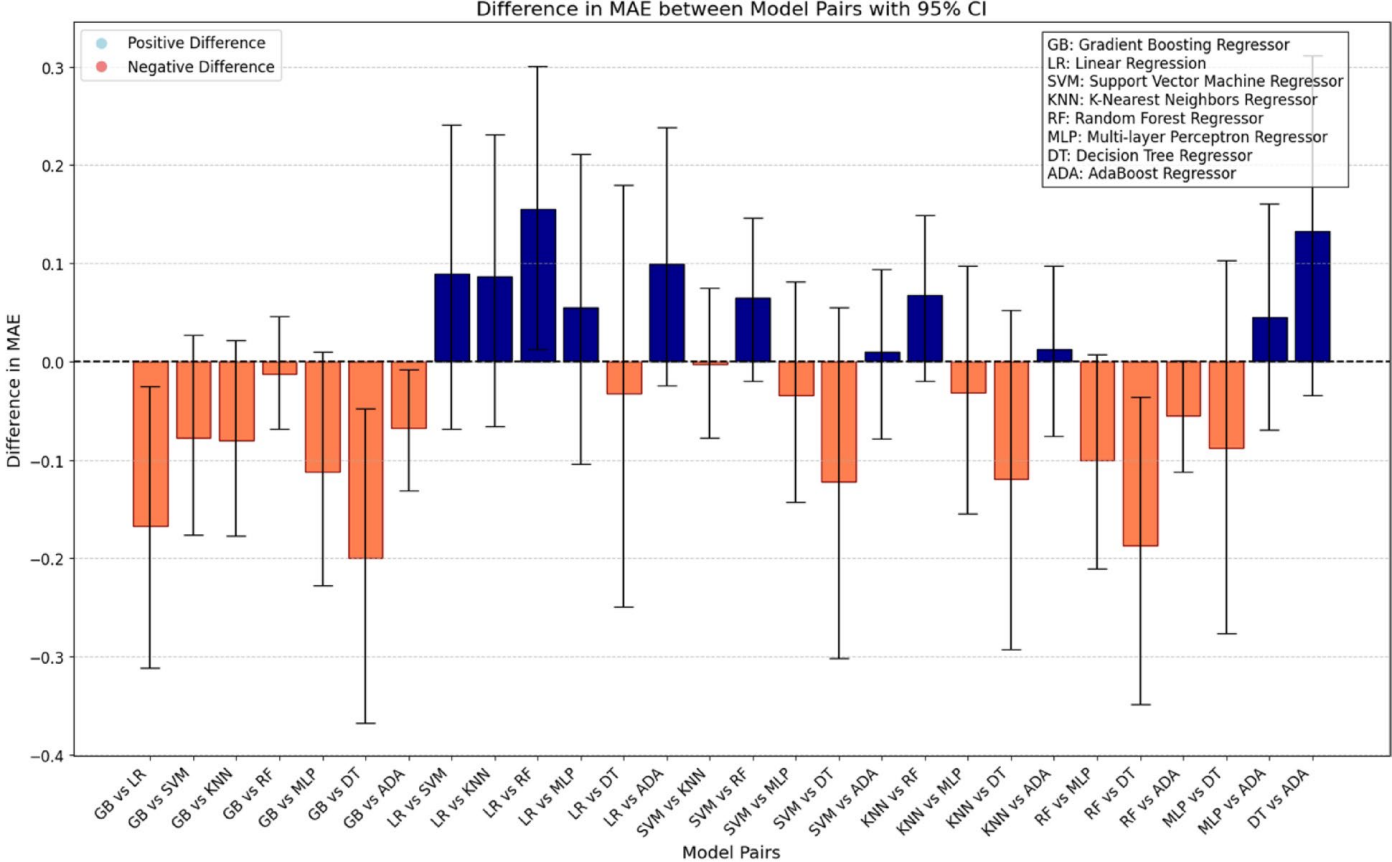
Difference in mean absolute error (MAE) between model pairs with 95% confidence intervals.

Pairwise comparisons of the mean absolute errors revealed distinct patterns of statistical significance among the evaluated models (Fig. 4). The decision tree model showed the poorest performance, with statistically significant differences when compared to the Gradient Boosting and Random Forest models. In contrast, the Gradient Boosting model achieved the best performance, with statistically significant differences compared to the Logistic Regression, AdaBoost, and Decision Tree models.

### Feature importance

**Fig. 5:**
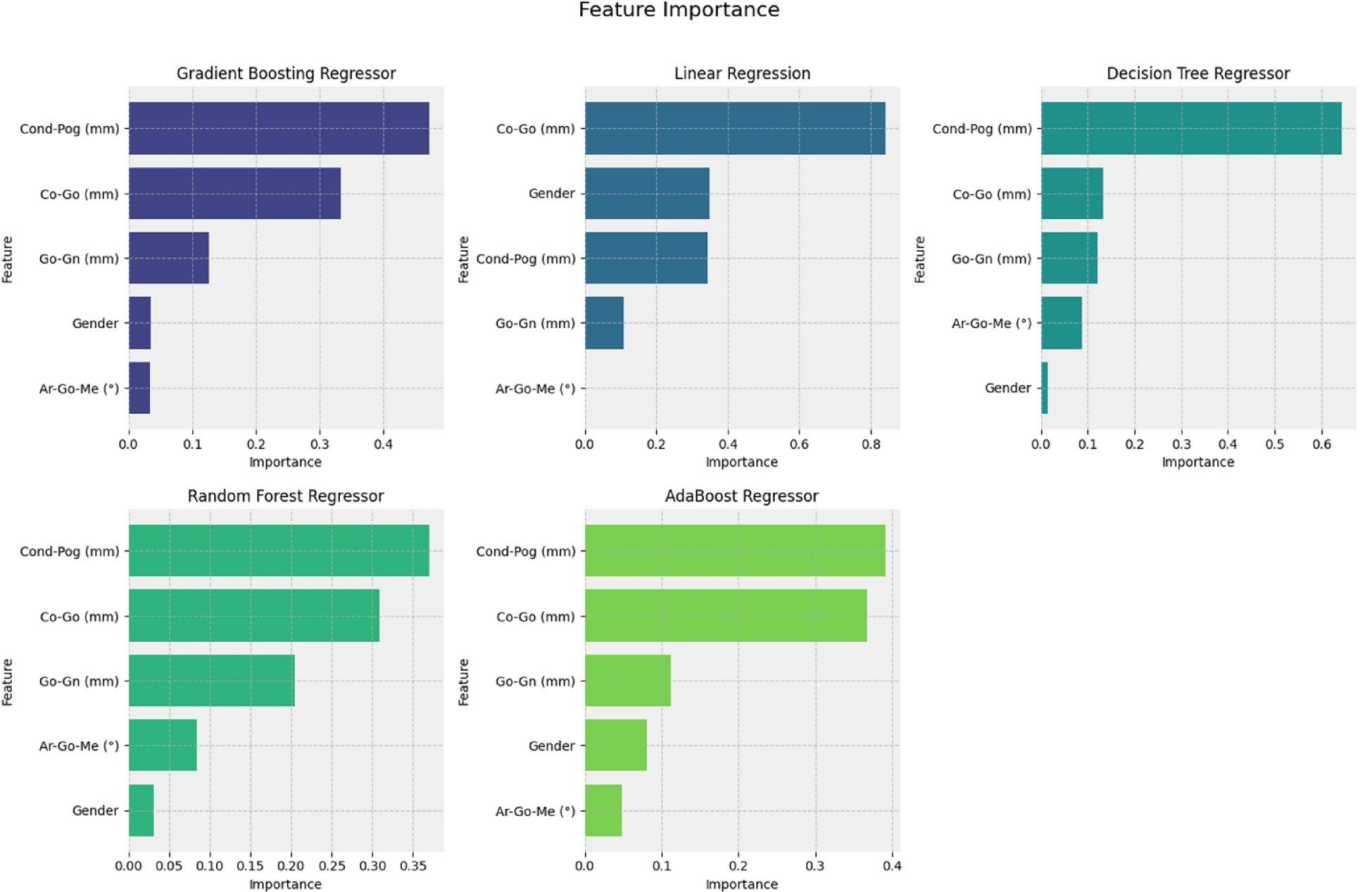
Feature importance ranking across different machine learning models.

Among the linear distances evaluated, total mandibular length (Co-Pog) demonstrated the highest predictive importance, followed by mandibular ramus height (Co-Go). The feature importance ranking is presented in Fig. 5.

## Discussion

The mandible plays a crucial role in forensic investigations, as its unique anatomical features, dental records, and bone structure can help identify individuals, determine age, and provide insights into cause of death. In age estimation, the mandible can play a crucial role in children and adolescents, not only due to its predictable growth patterns but also because of the sequential development of teeth. The current literature has repeatedly shown the importance of mandibular teeth to estimate age through dental age methods that assess the mineralization stages of developing permanent teeth using radiographic analysis. However, in certain forensic contexts, teeth may be absent from the mandible—whether due to ante-mortem loss, post-mortem damage, or developmental stage—limiting the applicability of traditional dental methods. In such cases, evaluating the use of mandibular dimensions for age estimation in growing individuals becomes particularly relevant. By exploring mandibular measurements and validating their accuracy across diverse populations, researchers and clinicians can enhance forensic identification methods and contribute to more precise age estimation in both living individuals and skeletal remains. Therefore, this study explores the application of mandibular morphometry combined with machine learning algorithms for age estimation.

In recent years, the application of AI in forensic medicine and odontology has grown substantially. A recent systematic review conducted by Singh et al. (2024) compiled studies that employed AI tools for age and sex estimation based on maxillofacial radiographs, including panoramic and lateral cephalometric images^20^. Although the review highlighted promising results, most of the analysed studies focused predominantly on sex determination or broad age group classification, with few studies aiming to precisely estimate chronological age. Furthermore, the majority of the reviewed studies relied on panoramic radiographs, which, despite providing a broad two-dimensional view of the maxillofacial region, present limitations for detailed morphometric analysis when compared to lateral cephalometric radiographs. Another important distinction concerns the age range of the studied populations: whereas previous studies primarily included teenagers and adults, our study focused exclusively on children and adolescents aged between 6 and 16 years, a developmental period characterized by intense skeletal changes. Additionally, while previous studies often incorporated dental parameters—such as the number of teeth or the presence of implants—we based our analysis solely on mandibular morphometric measurements, seeking greater applicability in forensic contexts where dental structures may be absent. It is also noteworthy that the predictive models developed in our study achieved a mean absolute error close to 1.5 years (18 months), which was lower than the 21 months reported by Back et al. using deep learning approaches^21^. Therefore, the use of mandibular morphometric measurements demonstrated the ability to produce more accurate age estimates in children and teenagers, expanding the applicability of forensic identification in challenging scenarios.

In forensic practice, a prediction error of approximately 1.5 years is often deemed clinically acceptable, particularly when age estimation relies on the assessment of a single skeletal indicator. The results obtained in this study is within this range, supporting their potential applicability in real-world forensic scenarios. Liversidge and Marsden (2010), in a study using third molars, reported mean absolute errors ranging from 1.45 to 1.97 years across six evaluated methods^22^. Previous studies have reported errors of 1.3 and 1.5 years for males and females, respectively, and 1.13 years using a polynomial and Bayesian approach^23,24^.

The dimensions of the mandible, particularly the height of the mandibular ramus and the gonial angle, have been extensively evaluated in the context of age estimation^7^. Specifically, the height of the mandibular ramus has been previously pointed as having a strong correlation with chronological age^25,26^. In our study, total mandibular length (Co-Pog) demonstrated the highest predictive importance, followed by the height of the mandibular ramus (Co-Go). This finding is particularly relevant, as the mandibular ramus typically remains intact even in extensively damaged skeletal remains, reinforcing its forensic applicability^27^. Furthermore, it is well established that the gonial angle undergoes morphological changes throughout life: it is initially obtuse during early developmental stages and gradually becomes more acute as the individual matures^28^. This transformation reflects the dynamics of skeletal growth, as the mandibular ramus tends to increase more in height than the mandibular body in length during growth, resulting in a reduction of the gonial angle^29^. These biological patterns highlight the potential of mandibular measurements for chronological age estimation, particularly in forensic cases where teeth may be absent or damaged. Thus, the investigation of mandibular morphometry combined with machine learning algorithms may represent a promising alternative for forensic age estimation in children and adolescents.

The application of AI for age prediction based on skeletal features offers significant advantages. AI-based models have demonstrated superior predictive performance and optimize analysis time compared to manual assessments by trained specialists. Integrating these tools into the assessment of bone structures can enhance accuracy, and support clinical decision-making, particularly in contexts with limited availability of specialized professionals^30^. Given that the mandible often remains intact even in severely compromised bodies^31^, mandibular dimensions analysed using machine learning algorithms can contribute to more precise age estimations in children and adolescents. In forensic practice, specific mandibular measurements can be directly extracted from radiographic images and applied to previously trained models, enabling rapid and standardized chronological age estimates. This approach enhances the speed and consistency of forensic examinations, especially in complex scenarios such as mass disasters or cases involving advanced skeletonization, thereby reducing the subjectivity associated with traditional assessments.

Finally, it is important to acknowledge some limitations of the present study. First, the sample consisted exclusively of German individuals with different skeletal malocclusions, which may limit the generalizability of the results to populations of different ethnic or geographic backgrounds. Additionally, one of the predictors used in the models was the sex of the individuals, which was included due to the distinct growth patterns observed between males and females. However, for practical application in real forensic contexts, the sex must be previously known or determined. Although sex can be reliably identified through methods such as DNA analysis, this additional step involves greater operational complexity and processing time, particularly in emergency forensic scenarios or situations with limited resources. Therefore, it is recommended that future studies validate these models in different populations and also explore alternative strategies for using or predicting the sex variable, aiming to broaden their applicability in forensic practice.

## Conclusions

The findings of the present study demonstrated that mandibular dimensions analysed by machine learning algorithms enable precise age estimation in children and adolescents, presenting mean absolute errors close to 1.5 years. Among the evaluated models, Gradient Boosting achieved the best predictive performance, demonstrating robustness and reliability for forensic applications. Additionally, total mandibular length (Co-Pog) and mandibular ramus height (Co-Go) were identified as the most significant predictors, reinforcing the forensic relevance of these anatomical measures. Nevertheless, the generalizability and practical applicability of the proposed predictive models may be influenced by population-specific characteristics and the availability of certain predictor variables in forensic contexts. Therefore, further studies are encouraged to validate these findings in diverse populations and explore alternative predictors to enhance applicability in various forensic scenarios.

## Data Availability

To ensure transparency and reproducibility, all scripts developed for the analysis are openly available at 10.5281/zenodo.15264847.
